# Thermal–Statistical Odd–Even Fermions’ Staggering Effect and the Order–Disorder Disjunction

**DOI:** 10.3390/e23111428

**Published:** 2021-10-29

**Authors:** Flavia Pennini, Angelo Plastino, Angel Ricardo Plastino

**Affiliations:** 1 Departamento de Física, Universidad Católica del Norte, Av. Angamos 0610, Antofagasta 3580000, Chile; fpennini@ucn.cl; 2 Departamento de Física, Facultad de Ingenieria, Universidad Nacional de Mar del Plata (UNMDP), CONICET, Mar del Plata 7600, Argentina; 3 Instituto de Física La Plata–CCT-CONICET, Universidad Nacional de La Plata, C.C. 727, La Plata 1900, Argentina; 4 CeBio-Departamento de Ciencias Básicas, Universidad Nacional del Noroeste de la Prov. de Buenos Aires (UNNOBA), CONICET, Junin 6000, Argentina; arplastino@unnoba.edu.ar

**Keywords:** odd–even staggering effect, statistical quantifiers, order–disorder

## Abstract

We review thermal–statistical considerations on the odd–even staggering effect (OES) in fermions. There is a well known OES in nuclear binding energies at zero temperature. We discuss here a thermal OES (finite temperatures) that establishes links with the order–disorder disjunction. The present thermal considerations cannot be found in the nuclear literature.

## 1. Introduction

A typical quantum microscopic phenomenon is that of the odd–even staggering (OES) of binding energies at zero temperature. The OES has been detected in variegated finite many-fermion systems. Examples are nuclei, ultra-small superconducting grains, and metal clusters [1].

The OES presents itself in the following fact: the binding energy of a system with an odd particle number is lower than the arithmetic mean of the energies of the two neighboring even-particle-number systems.

In atomic nuclei, the OES is commonly accredited to the presence of nucleon-nucleon pairing correlations [1]. A kindred mechanism has been suggested in the case of metallic grains [1]. In these two scenarios the concomitant Cooper pairing is well sketched with reference to a parity-number-conserving quasi-particle theory. Electrons in metals behave in a quite different fashion than that of nucleons in nuclei. However, the mechanism responsible for electronic and nucleons superconductivity, centered on an attractive residual interaction originating a many nucleon system endowed with correlations, is mostly the same in both scenarios [1,2,3,4]. The odd–even staggering in clusters is due to the Jahn–Teller phenomenon (see refs. [1,5,6], and references therein). The nuclear even–odd dissimilarities are also attributed to mean-field plus odd-nucleon blocking phenomena [7]. The nuclear binding energies (BE) were discovered long ago. In nuclei possessing an odd number of nucleons, BEs are smaller than the arithmetic mean of the BEs of their two neighbor-nuclei of even particle number. Fermion-pairing considerations are made responsible for this fact (amongst dozens of references, see Refs. [6,8,9,10] and references therein), where the OES phenomenon is attributed to such pairing [1,5,11].

### Our Goal

In this review, we wish to present a finite temperature thermal discussion of the OES in fermions, that is not necessarily related to the nuclear OES at zero temperature.

We appeal to several statistical quantifiers linked to the order–disorder disjunction. If *T* is low enough, the ensuing results will resemble those for the ground state. However, our main ideal is to relate staggering to order–disorder considerations.

We will do this by considering an analytically solvable fermionic-model that *does not possesses pairing interactions*. We will work at very low temperatures, so that results will resemble those for the ground state. We will see that odd–even differences appear in the thermal statistics of fermions-dynamics, with no reference to neither pairing interactions nor mean field effects. Contrary-wise, our thermal OES will be placed into a wider scenario of order–disorder considerations that will be quantified by appeal to a relatively new statistical notion, that of statistical complexity [12,13]. In the present treatment, “order” will be produced by fermion-fermion interactions while disorder will emerge by temperature *T* effects. Staggering will be exhibited below in the behavior of statistical quantifiers, the statistical complexity in particular.

The order–disorder game is described with reference to Gibbs’ canonical ensemble considerations. In them, the operating probability distribution becomes proportional to $\exp\left(-\beta \hat{H}\right)$, where $\hat{H}$ is the pertinent Hamiltonian (that one will use) and β the inverse temperature. This review is based on References [14,15].

## 2. The Model Discussed in This Review

Our present analytically solvable model is inspired by the celebrated Lipkin one [16], based on an SU(2) algebra. It yields accessible but not analytical exact solutions.

### 2.1. Present Model

We deal in this review with a simplified version of the Lipkin model advanced in Ref. [17] and utilized in [14,15].

### 2.2. Model’s Details

The models of [14,15,16,17] consider *N* fermions distributed between two (2N)-fold degenerate single-particle (sp) levels. Our two levels are separated by an energy gap ϵ. Two quantum numbers (denoted by the symbols mu and *p*) are ascribed to a general single particle state of the model. The first quantum number adopts two possible values identified by the value of a quantifier that we will call μ. The values are μ=−1 (lower level) and μ=+1 (upper level). The p−quantum number is often called a quasi-spin or pseudo spin one, and selects a specific micro-state pertaining to the *N*-fold degeneracy. The pair p,μ may be regarded as a kind of ”site” that is either full or empty. One has [14,15,16,17]

(1) N=2J,

*J* standing for an “angular momentum”. Following Lipkin et al. [16], we advance the so-called quasi-spin operators

(2) $${\hat{J}}_{+}=\sum_{p}{\hat{C}}_{p,+}^{†}{\hat{C}}_{p,-},$$

(3) $${\hat{J}}_{-}=\sum_{p}{\hat{C}}_{p,-}^{†}{\hat{C}}_{p,+},$$

(4) $${\hat{J}}_{z}=\sum_{p,\mu }\mu \;{\hat{C}}_{p,\mu }^{†}{\hat{C}}_{p,\mu },$$

(5) $${\hat{J}}^{2}={\hat{J}}_{z}^{2}+\frac{1}{2}\;\left({\hat{J}}_{+}{\hat{J}}_{-}+{\hat{J}}_{-}{\hat{J}}_{+}\right),$$

the eigenvalues of ${\hat{J}}^{2}$ adopting the form J(J+1).

The pertinent Hamiltonian [14,15,17] is ($\hat{I}$ is the unity operator and *J* the eigenvalue of $\hat{J}$).

(6) $$\hat{H}=\epsilon {\hat{J}}_{z}-V_{s}\left(\frac{1}{2}\left({\hat{J}}_{+}{\hat{J}}_{-}+{\hat{J}}_{-}{\hat{J}}_{+}\right)-J\hat{I}\right).$$

One usually sets either $V=V_{s}/\epsilon$ or ϵ=1. In addition,

(7) $$\hat{H}={\hat{J}}_{z}-V\left(\frac{1}{2}\left({\hat{J}}_{+}{\hat{J}}_{-}+{\hat{J}}_{-}{\hat{J}}_{+}\right)-J\hat{I}\right),$$

and the unperturbed ground state (gs) is the eigenstate for V=0. On account of Equation (1), the state

(8) $$|J,J_{z}\rangle =|J,-N/2\rangle ,$$

is endowed with an energy

(9) $$E_{o}=-N/2.$$

It is important to stress that doubly occupied p−sites are not permitted. $\hat{H}$ commutes with both ${\hat{J}}^{2}$ and ${\hat{J}}_{z}$.

Thus, the exact solution must belong to the *J*-multiplet containing the unperturbed ground state. These states can be cast in the fashion |J,M〉. Necessarily, one of them minimizes the total system’s energy. Its *M* value has to depend on the value of the coupling constant *V*.

A striking feature of the model of [17] is to be mentioned. As *V* grows from zero, $E_{o}$ does not at once vary. It keeps being $E_{o}$ until a critical V−special value is attained that equals 1/(N−1). We call this happening a level crossing. When this happens, the interacting ground state suddenly becomes |J,−N/2+1〉. If *V* continues increasing, new level crossings (pt) occur. That between $J_{z}=-k$ and $J_{z}=-k+1$ takes place at V=1/(2k−1). A pt-series ensues that ends when the interacting ground state becomes either $J_{z}=0$ ($V_{crit}=1$ for integer *J*), or $J_{z}=-1/2$ ($V_{crit}=1/2$ for odd *J*). In such instances, regardless the *J* one has [17]

(10) $$V_{crit}=1/2,$$

for half *J* and

(11) $$V_{crit}=1,$$

for integer *J*.

### 2.3. Finite Temperatures

Our Hamiltonian matrix is that of size (2J+1)×(2J+1), associated to the $J_{z}=-N/2$ multiplet, with N=2J [14,16]. Since we know all the Hamiltonian’s eigenvalues for this multiplet, we can immediately construct, given an inverse temperature β, the partition function in terms of a simple trace [14]:

(12) $$Z_{J}=\left(\exp\left(-\beta \hat{H}\right)\right),$$

and then the free energy F(J)

(13) $$F=-T\ln Z_{J}=-T\ln\left(\exp\left(-\beta \hat{H}\right)\right),$$

where, hereafter, we set the Boltzmann constant equal to unity. For each distinct *J* the trace is a simple sum over the $J_{z}$ quantum number *m*. Thus,

(14) $$Z\left(J\right)=\sum_{m=-J}^{m=J}\;\;\exp\left(-\beta E_{m}^{J}\right).$$

The pertinent energy $E_{m}^{J}$ is [17]:

(15) $$E_{m}^{J}=m-V\left[J\left(J+1\right)-m^{2}-J\right].$$

Consequently, the associated Boltzmann–Gibbs’ probabilities $P_{m}^{J}$ become [18]

(16) $$P_{m}^{J}=\frac{\exp\left(-\beta E_{m}^{J}\right)}{Z(J)},$$

for all m=−J,−J+1,…,J−1,J. Thus, the concomitant Boltzmann-Gibbs entropy becomes reads [18]

(17) $$S\left(J\right)=-\sum_{m=-J}^{m=J}\;\;P_{m}^{J}\ln P_{m}^{J}.$$

Note that the number of micro-states *m* is here:

(18) O(J)=2J+1,

which entails that the uniform probabilities $P(u_{J})$ that we need for building up the disequilibrium *D* discussed below is:

(19) $$P\left(u_{J}\right)=1/O\left(J\right).$$

## 3. Statistical Complexity *C* and Thermal Efficiency η

*C* is our central statistical quantifier [12,19,20,21,22,23,24]. Of course, the complexity-notion is pervasive in these days. All complex systems are usually connected to a certain conjunction of disorder/order and also to emergent phenomena. No acceptable by all definition exists. A famous definition for it was advanced by L. Ruiz, Mancini, and Calbet (LMC) [12], to which we appeal in this review. It is the product of an entropy *S* times a distance in probability space between an extant probability distribution and the uniform one. This distance is known as the disequilibrium *D*. Importantly enough, *D* is a measure of order. The larger *D* is the larger the amount of privileged states our system possesses. Our space of states is here a *J* multiplet. *D* adopts the form [12]

(20) $$D\left(J\right)=\sum_{m=-J}^{m=J}{\left[P_{m}^{J}-P\left(u_{J}\right)\right]}^{2},$$

and as stated, tells how large is the order in our system. More information about *D* can be consulted in Refs. [24,25]. The all important quantifier *C* adopts the appearance [12]

(21) C=SD.

### Thermal Efficiency

In our system we have one control parameter *V*. A perturbation in the control parameter, let us say from *V* to V+dV, will cause a change in the thermodynamics of the system. In the wake of Ref. [26], we define the efficiency η of our interactions as

(22) $$\eta \left(V;dV\right)=k_{B}\frac{dS}{dW},$$

where $k_{B}$ is Boltzmann’s constant, set =1 for convenience. dS and dW are, respectively, (i) the changes in entropy and (ii) the work done on (extracted from) the system caused by the dV variation. Thus, η(V;dV) represents the system’s decrease (growth) in uncertainty that ensues from each unit of work done. A small value of η indicates that much work is required to modify the extant order degree and vice versa for large η. In quasi-static processes one assumes that the system is always in equilibrium. It is demonstrated in Ref. [26] that:

(23) $$\eta \left(V;dV\right)=k_{B}\frac{\partial S}{\partial F},$$

involving Helmholtz’ free energy. The variation in free energy can be equaled with the work done on the system dF=dW [26]. Alternatively, we can think of η as the work needed (in changing *V*) so as to increase (η<0) or diminish (η>0) our information concerning the system. From still a different point of view, η is the work needed to augment (diminish) the degree of order in the system. We remark that:

(1) a negative η-task is one of growing order,
(2) a positive η-task is one of growing disorder.

## 4. Depicting Staggering in Our Fermion-Model

We will investigate the features of three statistical quantifiers: *D*, *C*, and η, all of them versus the number of fermions *N*. These three indicators clearly display odd–even staggering. The last one is original to our present task, and was not dealt with in references [14,15]. The other two quantifiers (*D*C and *C*) were dealt with in these references, but our present graphs are original too).

**Figure 1:**
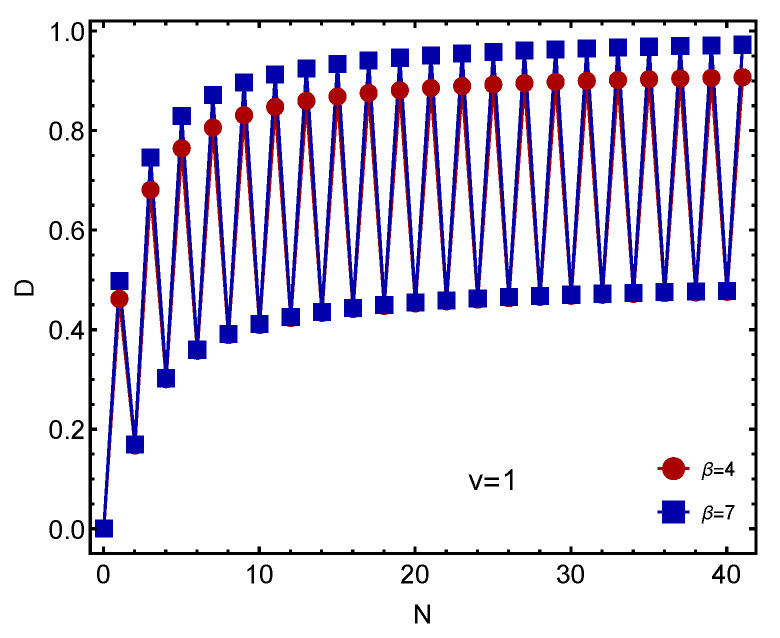
We depict *D* versus the the number of fermions *N* for two β values. One appreciates odd–even staggering. For even *N* the *D* values is equal for the two intervening β values.

Let us look first at Figure 1. Remember that *D* increases as in the system the degree of order augments. Examining Figure 1 one might be surprised because odd-fermion cases appear to be more ordered than even instances. We uncover here what interacting fermions actually do in the absence of (1) pairing interactions and (2) a mean field. This constitutes a novelty encountered by thermal–statistical quantifiers. Also, one sees in Ref. [14] that *D* increases with β and with *V*.

**Figure 2:**
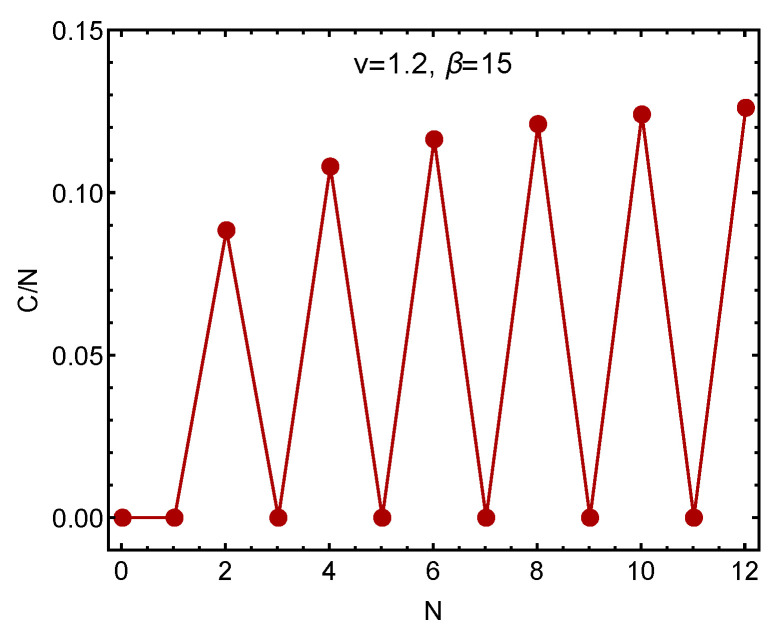
Normalized complexity C/N versus the number of fermions *N* at fixed β=15 and V=1.2. The staggering effect is evident.

Figure 2 displays C/N (a normalized ratio) versus *N*. Staggering is plainly noticeable. The complexity is larger for even than for odd *N* values. This is intuitively reasonable, as in Nature the behavior of the off-fermion (single closed shell nuclei for instance) dominates the system behavior at low excitation energies [27].

**Figure 3:**
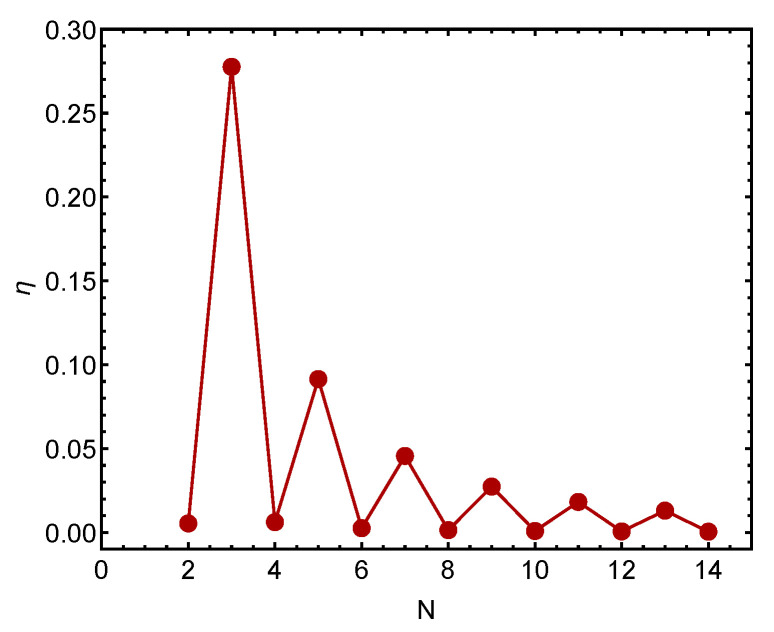
Thermal efficiency η versus *N*: The odd–even staggering is plainly visible. We see that it takes more work to change the coupling constant for odd than for even fermion numbers.

Figure 3 depicts the thermal efficiency η versus the fermion number. For N>14 we find that η becomes too small to comfortably fit into the Figure’s scale.

## 5. Conclusions

In the present review, we incontestably have seen the emergence of thermal–statistical odd–even staggering in interacting *N*-fermions collectives, as illustrated by the conduct, as a function of *N*, of:

- The thermal efficiency η,
- The disequilibrium.quantifier *D*, and
- The statistical complexity-quantifier *C*.

This staggering is part of an order–disorder environment. Interestingly enough, odd-fermion arrangements display a larger degree of order than even ones, as illustrated by the behavior of *D*.

## Data Availability

The data presented in this study are available in the article.
